# DEPDC1B, CDCA2, APOBEC3B, and TYMS are potential hub genes and therapeutic targets for diagnosing dialysis patients with heart failure

**DOI:** 10.3389/fcvm.2024.1442238

**Published:** 2025-01-08

**Authors:** Wenwu Tang, Zhixin Wang, Xinzhu Yuan, Liping Chen, Haiyang Guo, Zhirui Qi, Ying Zhang, Xisheng Xie

**Affiliations:** ^1^ Department of Nephrology, Nanchong Central Hospital Affiliated to North Sichuan Medical College, Nanchong, China; ^2^ Department of Nephrology, Guangyuan Central Hospital, Guangyuan, China; ^3^ Psychiatry Major, North Sichuan Medical College, Nanchong, China; ^4^ College of Clinical Medicine, North Sichuan Medical College, Nanchong, China

**Keywords:** maintenance hemodialysis, heart failure, RNA-Seq, regulatory network, WGCNA

## Abstract

**Introduction:**

Heart failure (HF) has a very high prevalence in patients with maintenance hemodialysis (MHD). However, there is still a lack of effective and reliable HF diagnostic markers and therapeutic targets for patients with MHD.

**Methods:**

In this study, we analyzed transcriptome profiles of 30 patients with MHD by high-throughput sequencing. Firstly, the differential genes between HF group and control group of patients with MHD were screened. Secondly, HF-related genes were screened by WGCNA, and finally the genes intersecting the two were selected as candidate genes. Machine learning was used to identify hub gene and construct a *nomogram model*, which was verified by ROC curve and RT-qPCR. In addition, we further explored potential mechanism and function of hub genes in HF of patients with MHD through GSEA, immune cell infiltration analysis, drug analysis and establishment of molecular regulatory network.

**Results:**

Totally 23 candidate genes were screened out by overlapping 673 differentially expressed genes (DEGs) and 147 key module genes, of which four hub genes (DEPDC1B, CDCA2, APOBEC3B and TYMS) were obtained by two machine learning algorithms. Through GSEA analysis, it was found that the four genes were closely related to ribosome, cell cycle, ubiquitin-mediated proteolysis. We constructed a ceRNA regulatory network, and found that 4 hub genes (TYMS, CDCA2 and DEPDC1B) might be regulated by 4 miRNAs (hsa-miR-1297, hsa-miR-4465, hsa-miR-27a-3p, hsa-miR-129-5p) and 21 lncRNAs (such as HCP5, CAS5, MEG3, HCG18). 24 small molecule drugs were predicted based on TYMS through DrugBank website. Finally, qRT-PCR experiments showed that the expression trend of biomarkers was consistent with the results of transcriptome sequencing.

**Discussion:**

Overall, our results reveal the molecular mechanism of HF in patients with MHD and provide insights into potential diagnostic markers and therapeutic targets.

## 1 Introduction

Chronic kidney disease (CKD) and heart failure (HF) often coexist. About 50% of HF patients have CKD at the same time (1, 2). Compared to the general population, patients with CKD have a higher prevalence of heart failure, which increases with deteriorating kidney function, especially in maintenance hemodialysis patients, with an incidence rate as high as 44% (2, 3). It is the second most frequent cardiovascular disease, accounting for 10.2% of all MHD-related cardiac deaths. The cardiovascular fatality rate for MHD patients is as high as 50% (4, 5). Like the general population, patients with MHD suffer form customary risk factors such as diabetes and hypertension, but it is inadequate to account for the increased incidence and mortality rate of HF in this group (6–8). Altered bone mineral metabolism, anemia and uremic toxin accumulation caused by renal function decline are closely related to HF (9). In addition, MHD treatment, which was originally used to maintain the patient's life, also increased the risk of HF, such as rapid changes in hemodynamics and electrolyte composition, myocardial injury caused by inadequate dialysis (6, 7, 10). Although numerous studies have comducted molecular research and employed bioinformatics technology to investigate HF in ordinary patients, there was a lack of information on the role of hub genes and potential molecular mechanisms for patients with MHD (11–13). This study aimed to investigate the biological processes implicated in pivotal genes of patients with MHD in the HF phase specifically via transcriptome sequencing, yielding novel perspectives the clinical diagnosis and treatment of such patients.

Due to varying degrees of volume overload affecting HF symptoms and signs, biomarkers and imaging examinations in MHD patients, were affected by volume overload to varying degrees, diagnosis of HF in this population remain challenging (14, 15). HF in patients with MHD was mainly treated by drugs and intensive hemodialysis, but traditional drugs did not effectively attenuate the progression of HF. In recent years, numerous studies had confirmed the efficacy of angiotensin receptor neprilysin inhibitors and Sodium-glucose cotransporter 2 inhibitors in controlling heart failure symptoms and improving myocardial remodeling. Unfortunately, most studies excluded the special population of patients with MHD, so there was still a lack of evidence on the efficacy and safety of drugs in this population (14, 15). Therefore, it was urgent to explore the characteristic genes closely related to HF in patients with MHD, in order to provide a better choice for the early diagnosis and treatment of HF in this population.

We used transcriptome sequencing technology to explore the transcriptome characteristics of HF in patients with MHD, screened hub genes, and the biological function of immune cells linked to hub genes is subsequently examined. After that, we performed real-time quantitative PCR (qRT-PCR) on peripheral blood samples of dialysis patients without HF and dialysis patients with HF human in order to validate our hypotheses. It provided a new reference for the diagnosis and treatment of HF in patients with MHD.

## 2 Material and methods

### 2.1 Sample preparation and processing

Peripheral blood mononuclear cell (PBMC) samples of 15 dialysis patients without heart failure (Normal) and 15 dialysis patients with heart failure (Case) human were collected for mRNA transcriptome sequencing. RNA was isolated and purified from the total samples using TRlzol (invitrogen, CA,USA) according to the manufacturer's protocol. NanoDrop ND-1000 (Wilminton, DE, USA) was used to detect purity and concentration of total RNA. The fragmented RNA was synthesized into cDNA by reverse transcriptase, and the cDNA was amplified and purified. Building the library and prepared for sequencing on the illumina Novaseq 6000 sequencing platform. After the inferior-quality reads were deleted, the cleanreads were aligned into the human genomic reference (GRCh38_gencode_v33) by HISATI (16).

### 2.2 Data processing and differential expression genes (DEGs) screening

DEGs between Case and Normal were identified using *R-package DESeq2* with the standard of *P*.value *<* *0.05* and *|log_2_FoldChange (FC)|* *>* *1*. The *R-package* “*ggplot2*” was utilised to create a volcano map, displaying differential genes with indication of the top 10 increased and decreased gene. All differential genes were sequenced according to *log_2_FC*, and expression heat map was drawn using the *R package* “*ComplexHeatmap*” (17).

### 2.3 Weighted gene co-expression network analysis (WGCNA)

The “WGCNA” package in R was used to construct the co-presentation network. First, the outlier samples were removed through the clustering analysis, and the weighted coefficient β was determined according to the scale-free network principle. Then, the modules were detected using the tree cutting algorithm and then calculate the correlation between the module and the disease. The module displaying the strongest correlation with the disease was designated as the pivotal module, and the genes within the module were identified as the pivotal module genes (18). The module's hub genes were overlaid with DEGs to obtain candidate genes.

### 2.4 Functional enrichment

To investigate the pathways and functions of the identified candidate genes, we conducted the Gene Ontology (GO) and Kyoto Encyclopedia of Genes and Genomes (KEGG) pathway enrichment analysis using the “*clusterProfiler*” package (19). Any result with a *P*.adjust < 0.05 was deemed statistically significant. The *R* package “*GOplot*” was used to plot the chordal graph in regard to the most significantly enriched GO items and KEGG pathway.

### 2.5 Acquisition of hub genes

To further identify hub genes, we used *Lasso* and *SVM-RFE* algorithms to screen candidates Using the “*Glmnet*” package of *R* language, *a binary logistic regression model of minimum absolute contraction and selection operator* (*Lasso*) was executed for candidate hub genes screening. Meanwhile, candidate genes were screened using recursive feature elimination by *support vector machine* for feature selection (*SVM-RFE*) via the “*caret*” package in the *R* language. The results of the two machine learning algorithms were intersected to obtain the hub genes, and the diagnostic value of the hub genes to gene was evaluated by *receiver operating characteristic* (*ROC*) curve. Moreover, to forecast the occurrence of heart failure following dialysis, we utilised the “*RMS*” package of the *R* language to create a *nomogram* featuring significant genes by *logistic regression*.

### 2.6 Gene set enrichment analysis (GSEA)

According to the median amount of hub gene expression, the samples were divided into two groups, one with high gene expression and the other with low gene expression. Difference analysis is performed by “limma”, sequencing genes according to their logFC values. The KEGG gene set was obtained from the MSigDB database (https://www.gsea-msigdb.org/gsea/msigdb/), and it was employed as the background set for GSEA. The top five pathways were visualized by enrichplot package of *R* (20).

### 2.7 Immune infiltration analysis

Enrichment scores for 28 immune infiltrating cell types were calculated using gene expression data based on ssGSEA algorithm within the *R* package “*GSVA*”. Heat maps were used to visualize the results. The difference between the Case and Normal groups was determined (*P*.value < 0.05), and the relationship between various immune cells and hub genes was assessed using Spearman correlation analysis through “*psych*”.

### 2.8 Competing endogenous RNA (ceRNA) regulatory network

The miRNAs targeting hub genes were predicted using miRDB and TargetScan database, and cross-over miRNAs were obtained. lncRNAs targeting cross miRNAs were predicted using miRanda and StarBase databases. The mRNA-miRNA-lncRNA regulatory network was constructed based on the obtained cross miRNAs, mRNAs, and lncRNAs (21).

### 2.9 Drug prediction

The *DrugBank* library was used to predict the small molecule drugs corresponding to hub genes, and the relationship between hub genes and drugs was visualized with *Cytoscape software (*22).

### 2.10 Real-time fluorescence quantitative PCR (Rt-qPCR)

Ten peripheral blood samples of patient with dialysis patients with and without heart failure were obtained from Nanchong Central Hospital with their knowledge and consent. This study was approved by Medical Ethics Committee of Nanchong Central Hospital. Total RNA has been purified with TRIZol (Thermo Fisher, Shanghai, CN), which was then used to inverse-transcribe mRNA into cDNA. Thereafter, qPCR analysis was carried out using 2xUniversal Blue SYBR Green qPCR Master Mix, and the PCR primer design is available in Supplementary Table S1. Glyceraldehyde 3-phosphate dehydrogenase (GAPDH) was utilised as the internal reference gene, whilst the expression levels of pivotal genes were measured using the 2^−*ΔΔ*Ct^ method.

### 2.11 Statistical analysis

All statistical analyses were conducted using *R* software. The Wilcoxon test was used to compare the differences between two groups, with a significance level of *P*.value < 0.05 applied unless otherwise specified.

## 3 Results

### 3.1 Differential expression analysis

**Figure 1:**
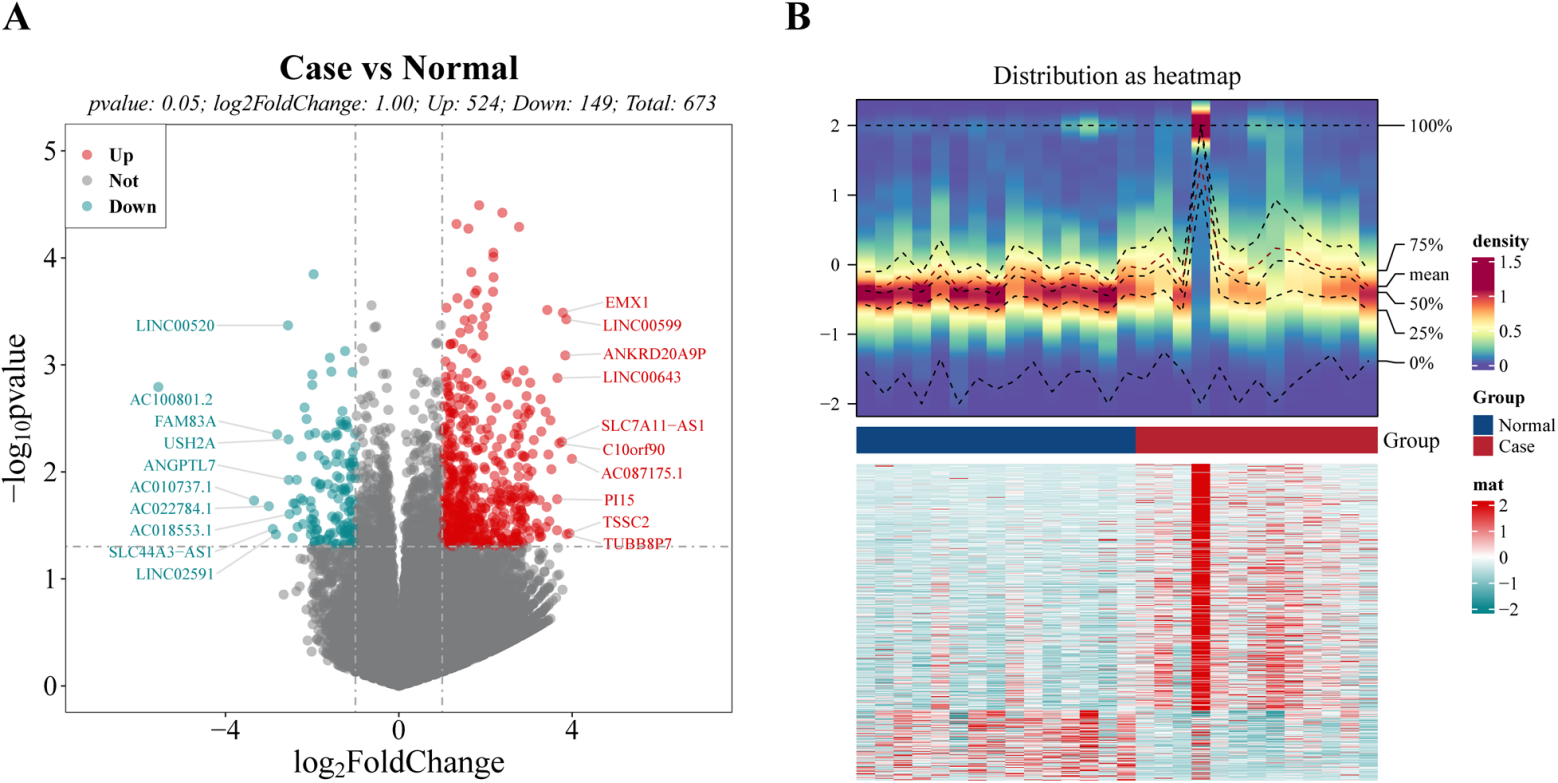
Identification of differential expression genes (DEGs) between case and normal groups. **(A)** Volcano plot of DEGs, with red dots signifying up-regulated genes, green dots representing down-regulated genes, and gray dots indicating undifferentiated genes. **(B)** Heat map of DEGs. The upper section presents a heat map illustrating the expression density of DEGs. It displays the lines representing the five quartiles and the mean expression levels. The lower section showcases a heat map depicting the expression of all DEGs.

The analysis revealed that a total of 673 DEGs meeting the thresholds of *P*.value < 0.05 and |log_2_FC| > 1 (Supplementary Table S2) were singled out, including 524 up-regulated genes and 149 down-regulated genes (Figure 1A). The top 10 up-regulated and down-regulated genes were labeled in the volcano map (Figure 1A). As can be seen in the heat map, there were significant differences between the disease and normal samples (Figure 1B).

### 3.2 Acquisition and enrichment analysis of candidate genes

**Figure 2:**
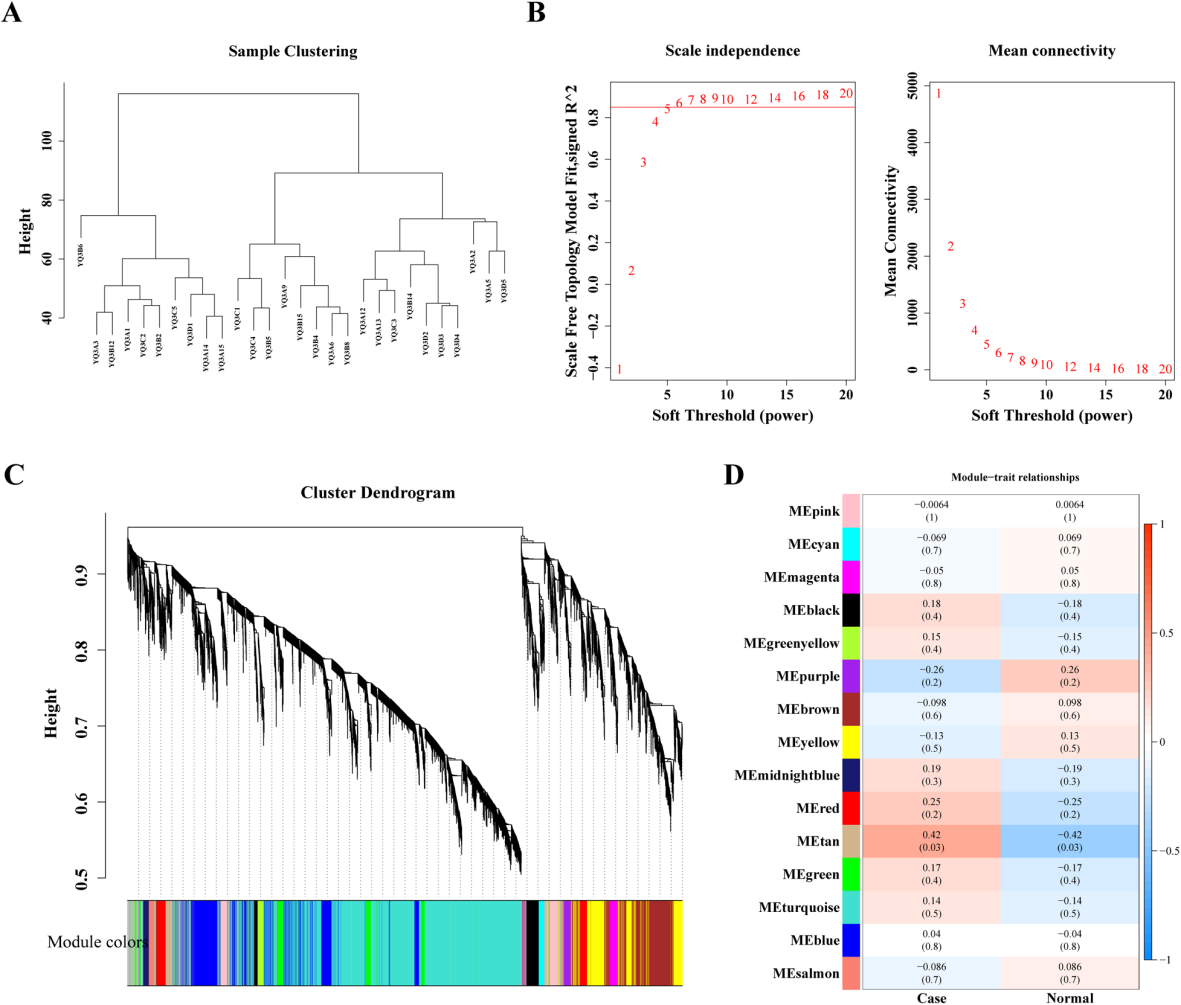
Weighted gene co-expression analysis (WGCNA) based on the 28 samples. **(A)** Sample hierarchical clustering. **(B)** Analysis of the scale-free index and mean connectivity for various softthreshold powers. **(C)** The gene dendrogram is generated using average linkage hierarchical clustering. The module assignment determined by the Dynamic Tree Cut algorithm is displayed below the dendrogram. **(D)** Positive and negative correlation coefficients of the WGCNA modules between case and normal group.

In order to screen genes associated with disease, 28 samples were analyzed by WGCNA. The clustering analysis results showed that there were no outliers among the 28 samples (Figure 2A), and the scale-free network was constructed by determining the soft threshold *β* = 6 (Figure 2B). These genes were clustered into several modules and partitioned with different colors. Finally, 15 different modules were identified (Figure 2C). In order to determine the correlation between phenotype and modules, a correlation analysis was performed. It was found that the tan module was highest positively correlated with Case (*Cor* = 0.42) (Figure 2D) containing 147 genes (Supplementary Table S3), which were related to the development of heart failure in dialysis patients.

**Figure 3:**
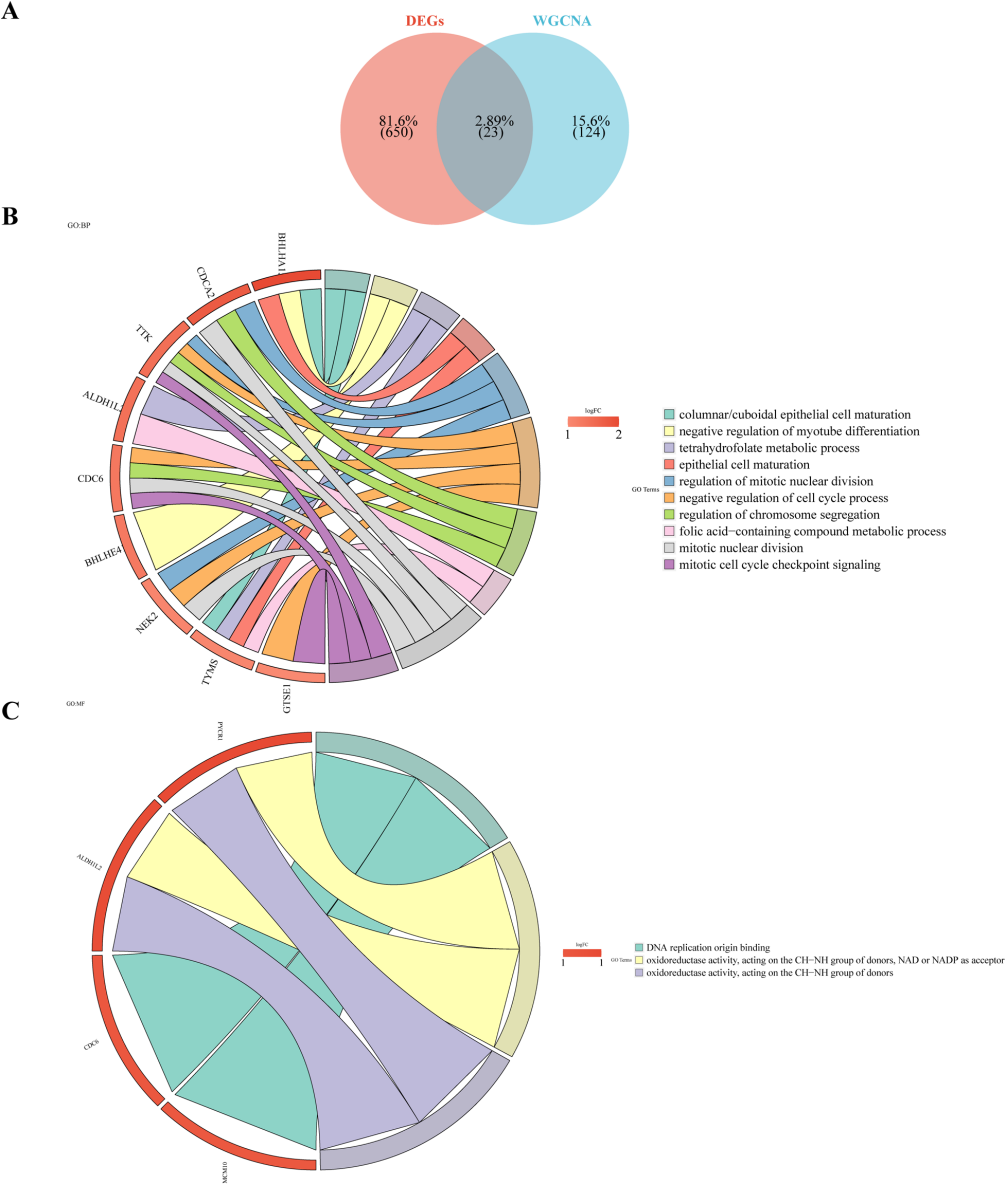
Candidate gene GO enrichment analysis. **(A)** Venn diagram illustrates the candidate genes by overlapping DEGs and case-related module genes. **(B)** Gene Ontology (GO) analysis of the candidate genes. **(C)** Kyoto Encyclopedia of Genes and Genomes (KEGG) analysis of the candidate genes.

Twenty-three intersection genes of DEGs and Case-related module genes was obtained as candidate genes, like KLHL14, DEPDC1B, CDCA2, ALDH1L2, APOBEC3B, TP73, TSHR, TTK, TYMS (Figure 3A). In order to analyze the biological pathways involved in candidate genes, candidate gene GO and KEGG enrichment analysis was carried out. After screening, the candidate genes were enriched in 37 GO biological functions (Supplementary Table S4), of which 9 candidate genes (BHLHA15, BHLHE41, ALDH1L2, CDCA2, CDC6, TYMS, NEK2, GTSE1, TTK) were enriched in the top 10 biological processes (GO:BP), such as cylindrical/cubic epithelial cell maturation, negative regulation of myoduct differentiation, tetrahydrofolate metabolism, epithelial cell maturation, mitotic cell division regulation (Figure 3B). Four candidate genes (ALDH1L2, PYCR1, CDC6, MCM10) were enriched in three molecular functions (GO:MF), including DNA replication starting point binding, REDOX activity—donor NAD or NADP acting on the CH-NH group as a receptor, REDOX activity—acting on the donor CH-NH group (Figure 3C). For example, TYMS was involved in the maturation of columnar/cubic epithelial cells, tetrahydrofolate metabolism, epithelial cell maturation, and the metabolism of folate-containing compounds. Additionally, these genes were found to be involved in One carbon pool by folate, Cell cycle, Maturity onset diabetes of the young, Antifolate resistance, Circadian rhythm, p53 signaling pathway (Figure 3D).

### 3.3 Hub genes in the process of heart failure after dialysis

**Figure 4:**
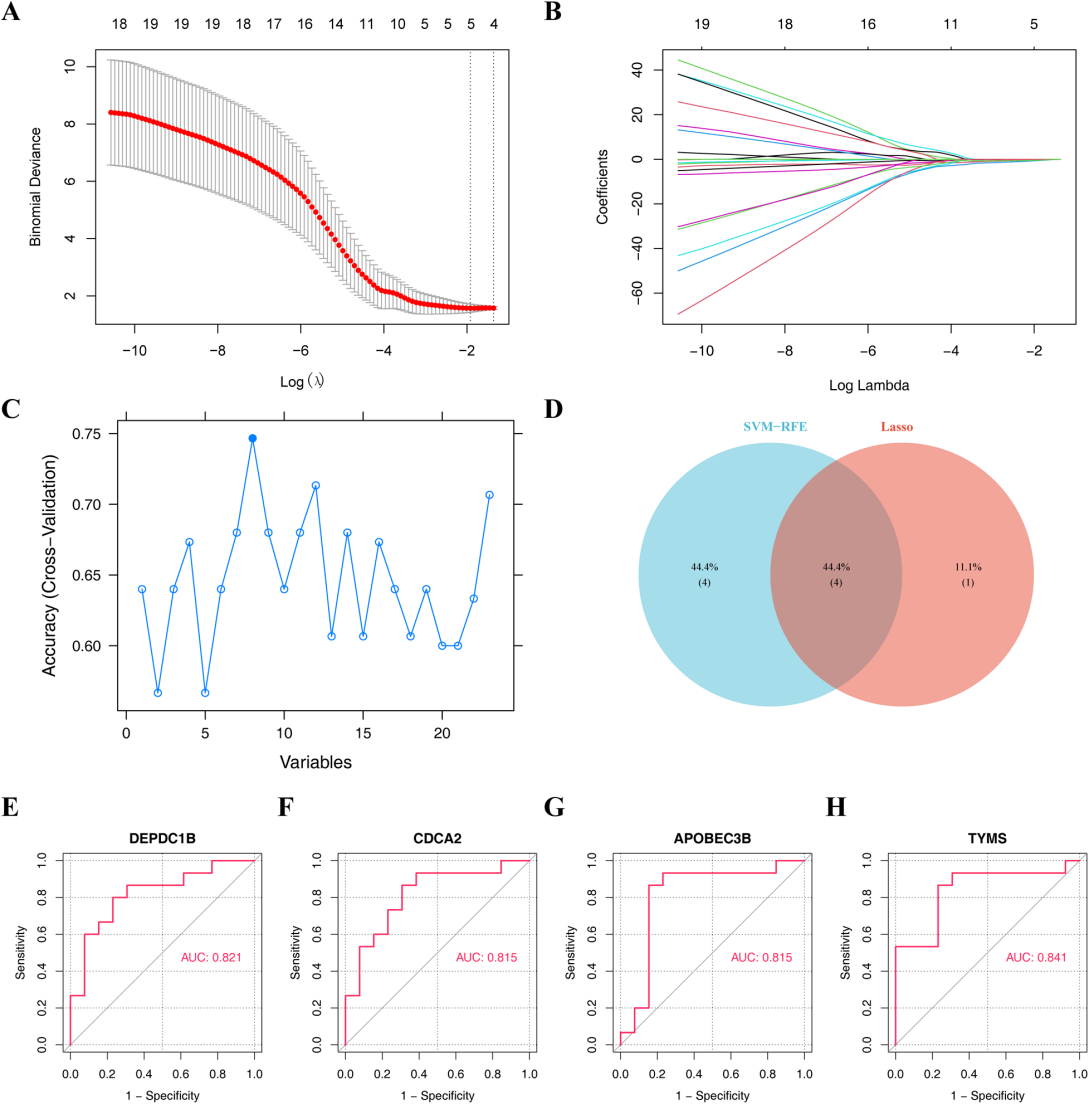
Machine learning algorithms screen hub genes. **(A,B)** Obtaining five characteristic genes through *Lasso* algorithm. **(C)** Obtaining eight characteristic genes by *SVM-RFE* algorithm. **(D)** Venn diagram illustrates the hub genes by overlapping both machine learning algorithms. Receiver operating characteristic (ROC) curve of **(E)** DEPDC1B, **(F)** CDCA2, **(G)** APOBEC3B, and **(H)** TYMS.

To identify hub genes in dialysis patients with heart failure, the *Lasso* and *SVM-RFE* algorithms have been used for selection. Five characteristic genes were obtained using *Lasso* algorithm, including DEPDC1B, CDCA2, RGS16, APOBEC3B and TYMS (Figures 4A,B). Eight characteristic genes, TYMS, CDC6, DEPDC1B, APOBEC3B, CDCA2, GTSE1, TTK and KLHL14, were obtained by *SVM-RFE* algorithm (Figure 4C). The results of *Lasso* and *SVM-RFE* were combined to obtain four hub genes (DEPDC1B, CDCA2, APOBEC3B, and TYMS) (Figure 4D).

**Figure 5:**
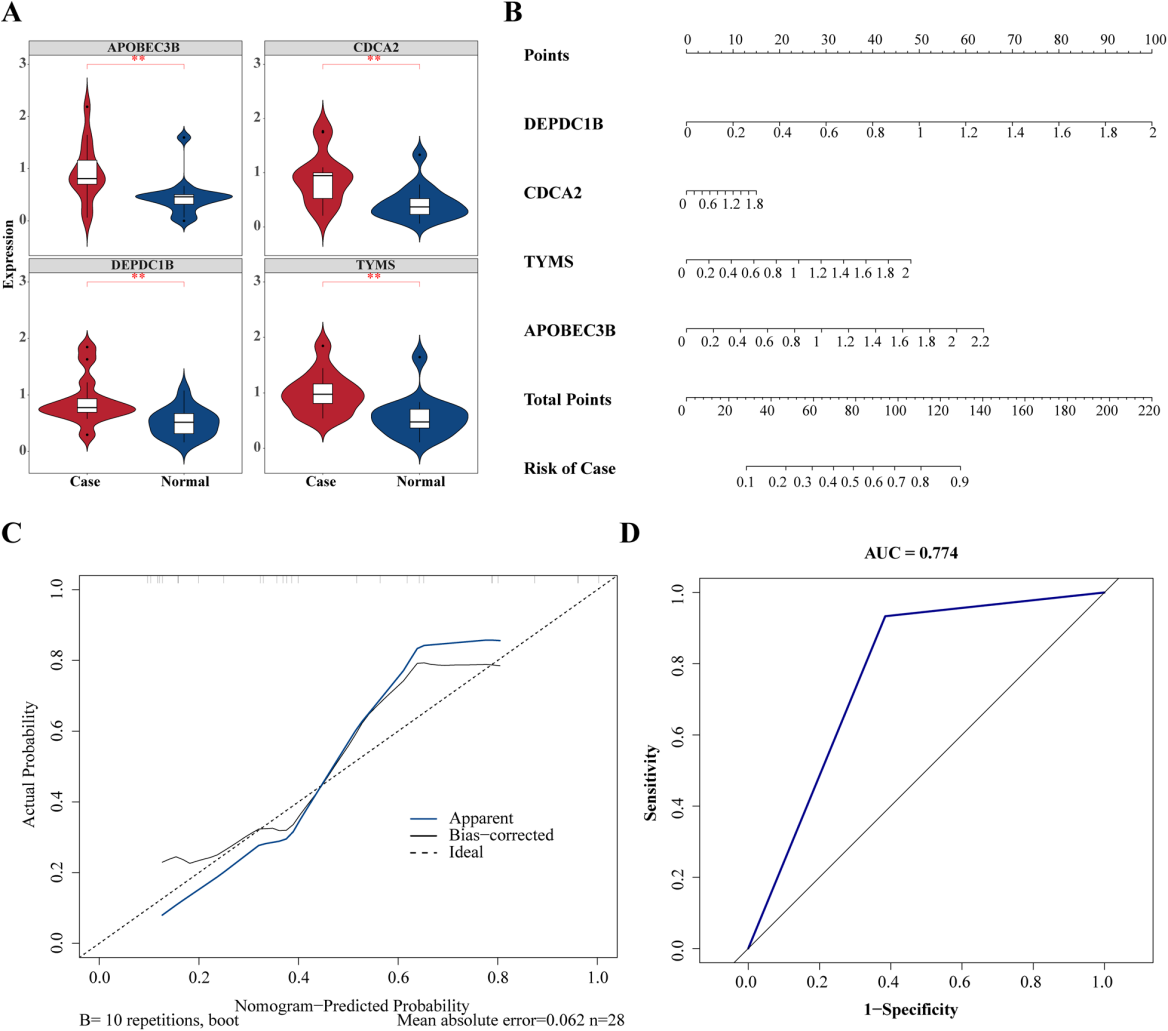
Validation of diagnostic value of hub gene in heart failure. **(A)** Expression level analysis of hub genes between case and normal groups. *P < 0.05, **P < 0.01, ***P < 0.001. **(B)** Construction of hub genes related *nomogram*. **(C)** *Calibration curves* to evaluate the association between actual probability and predicted probability. **(D)** *Receiver operating characteristic* (*ROC*) curve to evaluate the sensitivity and specificity of *nomogram*.

In order to obtain the diagnosis value of four hub genes for Case, *ROC curves* of single genes were drawn, and it was found that all four hub genes had suitable diagnostic value for dialysis with heart failure (*AUC* > 0.8) (Figures 4E–H). Besides, the expressions of DEPDC1B, CDCA2, APOBEC3B, and TYMS were considerably greater in Case samples compared to Normal samples (Figure 5A).

To forecast the likelihood of heart failure following dialysis, a *nomogram* was constructed based on four hub genes (Figure 5B). Calibration curves showed that the *nomogram model* could predict heart failure caused by dialysis (Figure 5C). Based on the ROC curve drawn on the *nomogram*, the *AUC value* of the *ROC curve* was 0.774, demonstrating that the *nomogram* model exhibited significant diagnostic value (Figure 5D).

### 3.4 GSEA of hub genes

**Figure 6:**
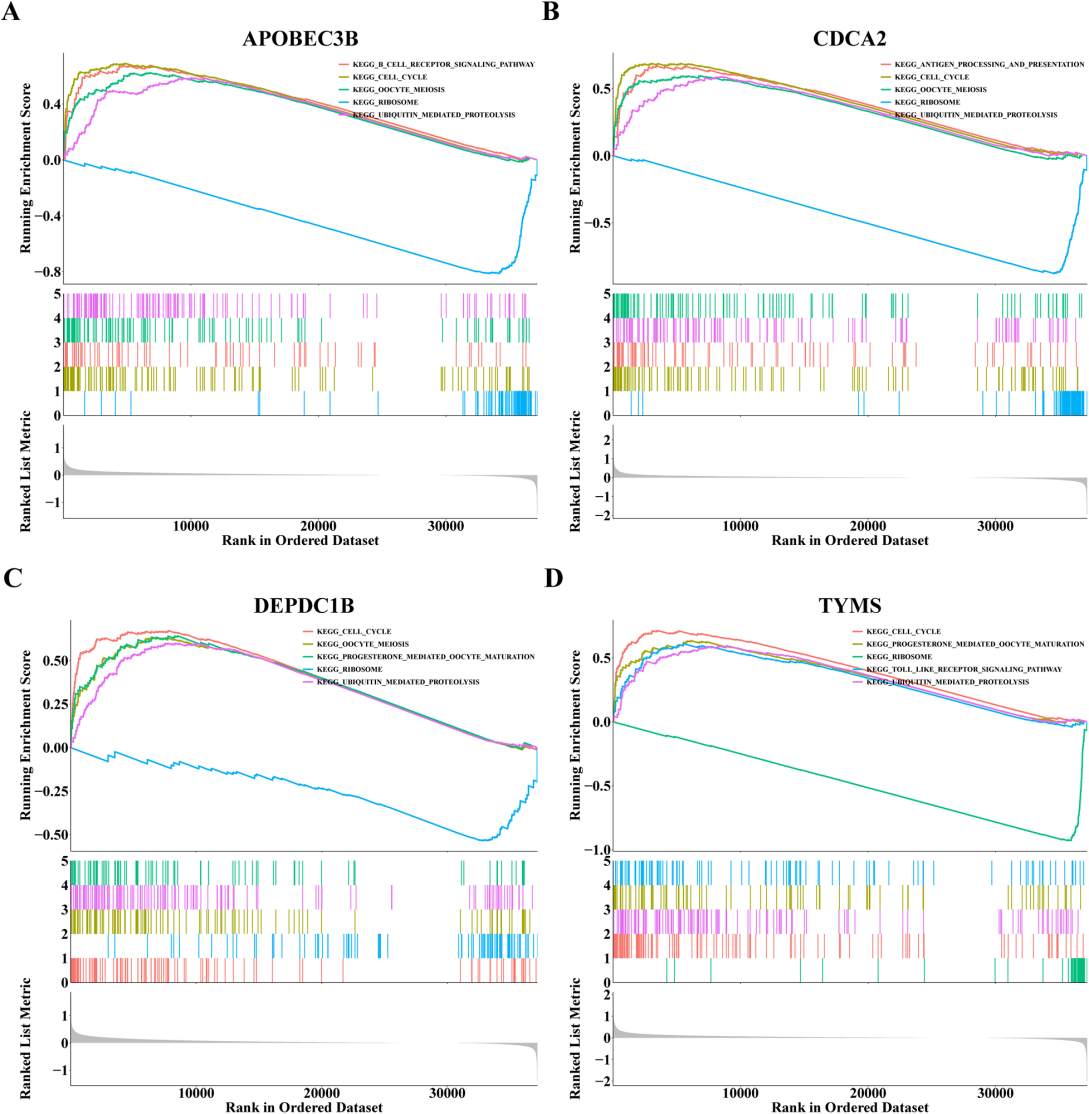
Gene set enrichment analysis (GSEA) results of **(A)** APOBEC3B, **(B)** CDCA2, **(C)** DEPDC1B and **(D)** TYMS.

To investigate the signal pathway of notable enrichment of hub genes, GSEA was carried out. The results showed that APOBEC3B, CDCA2, TYMS and DEPDC1B all significantly affected the ribosome and cell cycle, Oocyte meiosis and ubiquitin mediated protein breakdown process (Figure 6). In addition, APOBEC3B also affected the B-cell receptor signaling pathway, and CDCA2 had a significant impact on the process of processing and presenting antigens, whereas TYMS had a significant effect on the toll-like receptor signaling pathway (Figure 6). These findings suggest that four hub genes may influence the onset and progression of dialysis patients with heart failure through these pathways.

### 3.5 Relationship between hub genes and immune cells

**Figure 7:**
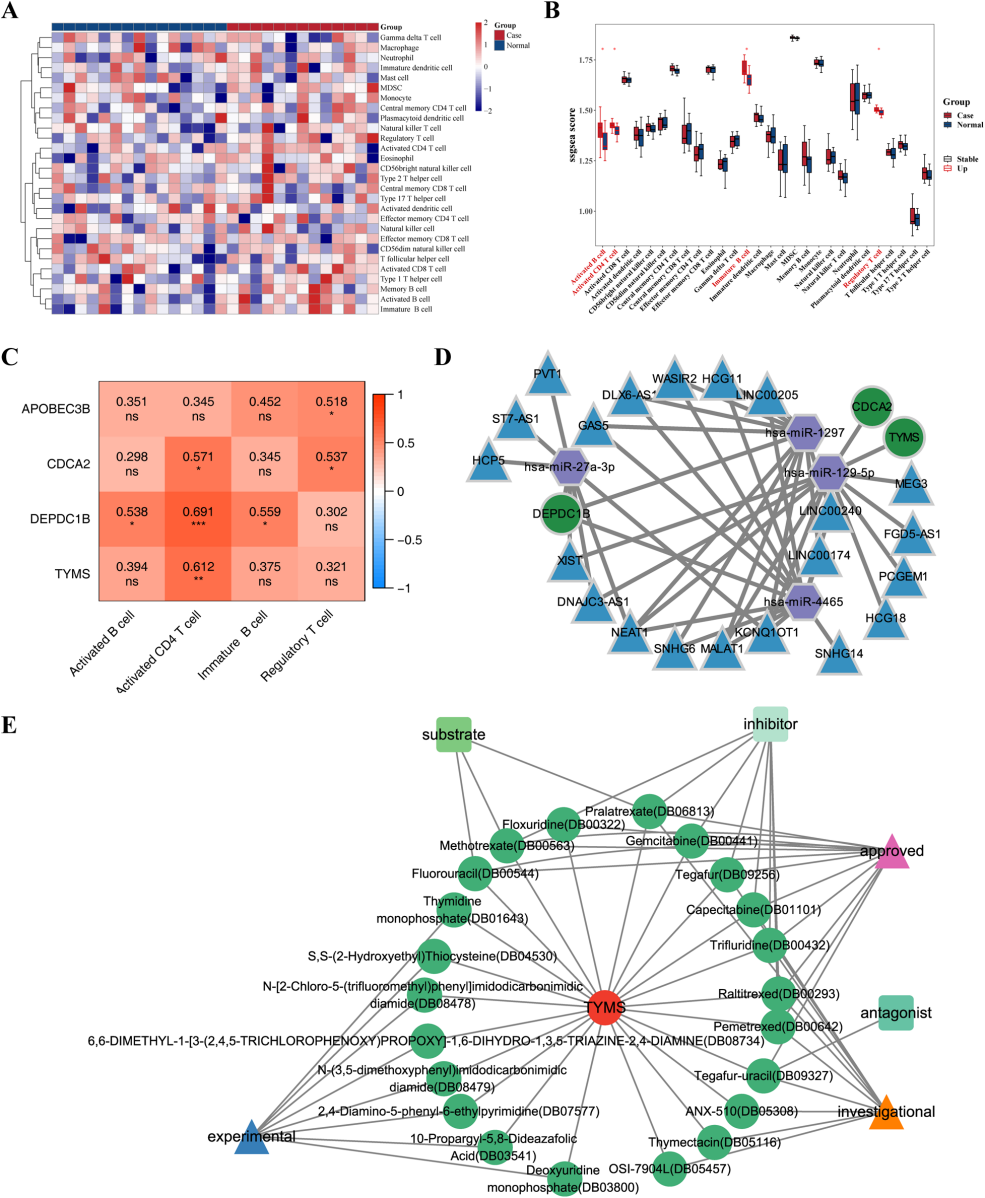
Relationship between hub genes and immune cells & CeRNA regulatory network of hub genes. **(A)** Heat map of enrichment fraction of 28 types of immune infiltrating cells between case and normal samples. **(B)** Box plot illustrate the difference in enrichment fraction of 28 types of immune infiltrating cells between case and normal samples. *p < 0.05. **(C)** Heat map illustrate the correlation between different immune infiltrating cells and hub genes. *p < 0.05, **p < 0.01, ***p < 0.001, ns: *p* > 0.05. **(D)** Competing endogenous RNA (ceRNA) regulatory network of hub genes is depicted, with mRNA represented in green, miRNA in purple, and lncRNA in blue. **(E)** Drug prediction network of hub genes, where red circles represent genes, green circles represent drugs, triangles represent drug states, and quadrilaterals represent drug types.

The heat map displayed the enrichment fraction of 28 types of immune infiltrating cells between Case and Normal samples (Figure 7A). Four kinds of immune infiltrating cells (Activated B cell, Activated CD4*^+^ T* cells, Immature B cell and Regulatory *T* cell) in Case samples were considerably greater than that in the Normal samples (Figure 7B). Spearman correlation was analyzed between 4 different immune infiltrating cells and 4 hub genes. The results showed that all 4 hub genes showed positive correlations with the 4 differential immune cells, in which CDCA2 DEPDC1B, and TYMS showed the highest correlation with activated CD4+ *T* cells (*cor* = 0.571, 0.691, 0.612), while APOBEC3B showed the highest correlation with regulatory *T* cells (*cor* = 0.518) (Figure 7C). Through immune infiltration analysis, it was found that four hub genes can interact with Activated B cells, Activated CD4+ *T* cells, Immature B cells and Regulatory *T* cells to further affect dialysis patients with heart failure.

### 3.6 CeRNA regulatory network of hub genes

The miRNAs of hub genes were predicted using miRDB and TargetScan database respectively, and four miRNAs (*hsa-miR-1297*, *hsa-miR-4465*, *hsa-miR-27a-3p*, *hsa-miR-129-5p*) were obtained after intersection, of which hsa-miR-1297, hsa-miR-4465, and hsa-miR-27a-3p targeted DEPDC1B, and hsa-miR-129-5p targeted TYMS and CDCA2. Unfortunately, no miRNA targeting APOBEC3B was retrieved. The intersection lncRNAs were predicted by miRANDA and StarBase databases, and 21 intersection lncRNAs were obtained. The ceRNA regulatory network was plotted by Cytoscape software, which showed that DEPDC18 was regulated by *hsa-miR-4465*, *hsa-miR-1297* (Figure 7D); both CDCA2 and TYMS were regulated by *hsa-miR-129-5p* (Figure 7D).

### 3.7 Drug prediction based on hub genes

The DrugBank database was used to predict the small molecule drugs corresponding to 4 hub genes. Among the four hub genes, only TYMS predicted multiple small molecule drugs. A total of 24 small molecule drugs targeting TYMS were predicted, including Raltitrexed, Floxuridine, Pemetrexed, Capecitabine, Fluorouracil (Supplementary Table S5), contributing to the development of new therapeutic targets for dialysis patients heart failure. The relationship between TYMS and small molecule drugs was visualized by Cytoscape software (Figure 7E).

### 3.8 Verification of gene expression

**Figure 8:**
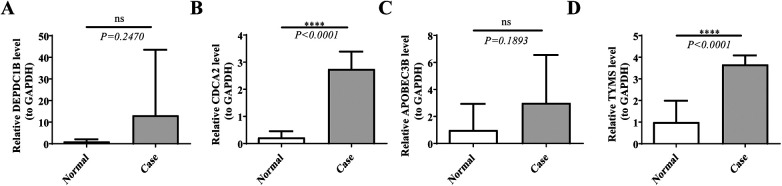
The expression levels of hub genes. The expression levels of **(A)** DEPDC1B, **(B)** CDCA2, **(C)** APOBEC3B, and **(D)** TYMS were analyzed in both case and normal samples by real-time fluorescence quantitative PCR (RT-qPCR). *p < 0.05, **p < 0.01, ***p < 0.001, ****p < 0.0001, ns: *p* > 0.05.

The RT-qPCR results had revealed that the expression levels of both CDCA2 and TYMS were significantly up-regulation in the Case samples compared to Normal samples, which were consistent with the results of bioinformatics analysis (Figure 8; Supplementary Figure S1). In addition, the expression of DEPDC1B and APOBEC3B was not significant between the Case and Normal groups, which might be due to the small sample size. In the future, we will keep focusing on them.

## 4 Discussion

Due to the considerably higher incidence of HF in patients with MHD compared to the general population, the combination of HF and MHD results in a significant rise in hospitalization rates, risk of mortality and economic burden (2, 4, 5). Clinical data demonstrate that traditional risk factors, uremia and dialysis-related risk factors contribute to the occurrence and development of HF in patients with MHD (14, 15). This raises the hypothesis that genes may play a critical role in the pathogenesis (14, 15). More and more evidence shows that HF is a polygenic disease with a large number of genetic components and high heritability (23). Although the diagnosis and treatment of HF has made great progress in recent years, the diagnosis and treatment of HF in Patients with MHD is still difficult because almost all studies exclude Patients with MHD (14, 15).

This study was the pioneering effort to investigate the biological processes underlying the cruial genes implicated in the HF pathogenesis of MHD patients based on transcriptome sequencing. Through a series of bioinformatics methods (WGCNA, Protein-Protein Interaction) and machine learning algorithms (*SVM-RFE* and *Lasso* regression analysis), four hub genes (APOBEC3B, CDCA2, TYMS and DEPDC1B) were finally identified, and their hub genes and molecular pathways were further analyzed. Subsequent immune infiltration analysis found that CDCA2, DEPDC1B, and TYMS are closely related to *CD4^+^ T* cells activation and myocardial infiltration and injury, which helps us to further understand the immune-related pathogenesis of HF in the context of MHD. Through qPCR detection, our clinical specimens verified the high expression of four hub genes in HF of MHD patients. Through literature review, we found that the above four genes have not been deeply studied in HF of MHD patients so far. Therefore, the results of this study will help to broaden the horizons of their biological functions and molecular mechanisms, and provide new insights for the clinical diagnosis and treatment of HF in MHD patients.

Several studies shown that 3 hub genes (DEPDC1B, APOBEC3B, CDCA2) were linked to HF in the MHD setting. DEPDC1B (DEP domain protein 1B), located on chromosome 5 (5q12.1), is a gene that encodes a protein involved in the regulation of cell growth and division. The DEPDC1B protein is a member of the DEP (Dishevelled, Egl-10, and Pleckstrin) domain-containing protein family and has been found to play a role in various cellular processes such as the cell cycle and cell proliferation. The function of DEPDC1B is not fully understood, but some studies have shown that it plays an important role in regulating cell mitosis, transcription and tumorigenesis (24, 25). It contained two conserved domains: DEP domain and RhoGAP domain. The DEP domain was not only a membrane anchor protein, but also negatively interacted with charged phospholipids located on the membrane, thereby activating the Wnt signaling pathway (26). Recent evidence suggested that Wnt/β-catenin-mediated cardiomyocyte hypertrophy and cardiac fibroblast activation may eventually progress to HF (27, 28). However, further investigation was required to determine the precise significance of DEPDC1B in MHD patients with HF. Our study found four miRNAs that predicted hub genes by establishing a ceRNA network, namely *hsa-miR-1297*, *hsa-miR-4465*, *hsa-miR-27a-3p*, and *hsa-miR-129-5p*, where *hsa-miR-4465* and *hsa-miR-27a-3p* are involved in the targeted regulation of DEPDC1B. Previous studies have shown that *hsa-miR-4465* plays a key role in regulating glucose metabolism, glutamine metabolism, autophagy and cancer progression in various cell types (29), while *miR-27-3p* inhibits adipogenesis by inhibiting PPARγ (30, 31). A large number of studies have shown that glucose and lipid metabolism disorders are significantly associated with the formation of HF (32, 33). Therefore, *miR-4465* and *hsa-miR-27a-3p* may induce the occurrence of HF in MHD patients by affecting DEPDC1 B and inducing glucose and fat metabolism disorders. Future research is expected to further explore this field. Studies indicated that the APOBEC3B (Apolipoprotein B mRNA Editing Enzyme Catalytic Subunit 3B) gene was a member of the APOBEC family of proteins, which are cytidine deaminases involved in innate immunity and antiviral defense, and it primarily caused cytosine mutations, resulting in DNA/RNA alterations (34). Daniela's recent study found that the typical and atypical nuclear factor-kappa B (*NF-κB*) pathway could mediate the high expression of APOBEC3B in immune or tumor cells (35). Several studies indicated that, in activated B cells, reactive oxygen species (*ROS*)—mediated oxidative stress promotes the progression of cardiomyopathy to HF, and *NF-κB* was a key mediator of oxidative stress (36, 37). Finally, *NF-κB* was translocated to the nucleus and triggers inflammatory genes, which promoted the occurrence of congestive heart failure (38, 39). However, more detailed and broader studies were still needed to comprehensively evaluate the specific mechanism of the *NF-κB*/APOBEC3B pathway in MHD patients with HF. CDCA2 (Cell Division Cycle Associated 2) is a gene that plays a crucial role in cell cycle regulation and cell division. It encodes a protein known as CDCA2, which is involved in coordinating various processes during cell division. Currently, most studies of CDCA2 focused on cancer, revealing that CDCA2 promotes tumor growth and inhibits apoptosis by regulating BRCA1-NRF2/p53-PUMA in liver cancer (40, 41), or by activating the CCAD1/AURKA signaling pathway to promote melanoma proliferation and migration (42, 43). In addition, Zhen's recent study found that the expression of CDCA2 expression was closely related to p53 signaling and apoptosis in hepatocellular carcinoma cells (Huh-7) (41). There was also evidence that activation of the p53 signaling pathway leads to apoptosis and cardiac fibroblast activation/proliferation, which ultimately induces the occurrence of HF (44, 45). Interestingly, our study found that TYMS and CDCA2 received hsa-miR-129-5p targeting regulation. Recent studies have shown that miR-129-5p is involved in the regulation of the syndecan signaling pathway and the level of IL-4, which affects cardiac fibroblast differentiation and collagen cross-linking, ultimately leading to cardiac remodeling (38, 46, 47). On the other hand, it was also involved in the regulation of Toll-like receptor (*TLR*)-4, which was highly expressed in the heart, binds to endogenous ligands and activates cascade reactions (38). Although conventional biomarkers (N-terminal pro-B-type natriuretic peptide and B-type natriuretic peptide) have shown excellent performance in detecting CHF patients, Reza's study found that *hsa-miR-129-5p* has potential added value with circulating natriuretic peptides in the diagnosis of different types of CHF (38). The specific mechanism of miR-129-5p mediated CDCA2 and CDCA2 involved in P53 signaling pathway in cardiomyocytes deserves further study.

Unlike the above 3 genes, no evidence of TYMS was been found in HF studies. TYMS (Thymidylate Synthase) is an enzyme that plays a pivotal role in DNA synthesis and replication. It is involved in catalyzing the conversion of deoxyuridine monophosphate (dUMP) to deoxythymidine monophosphate (dTMP), which is a nucleotide necessary for DNA replication and repair. Currently, there were few studies on TYMS gene polymorphism and HF. TYMS was a folate-dependent essential enzyme that produces the only intracellular *de novo* source of dTMP required for DNA synthesis and repair (48). Current studies shown that elevated TYMS mRNA and protein levels were associated with anti-cancer drug resistance or worse clinical prognosis in a variety of hematological and solid tumors (49–51). This study explored the association between TYMS gene polymorphisms and HF in the context of MHD for the first time. We will continue to pay attention to its mechanism, which is also our future research direction.

Further immune infiltration analysis showed that there were 4 different immune cells in the MHD combined HF group and the control group. The results of our correlation analysis demonstrated a positive correlation between the following immune cells Activated B cell, Activated *CD4^+^ T* cells, Immature B cell and Regulatory *T* cell. Among these correlations, we found that CDCA2, DEPDC1B and TYMS were moderately correlated with Activated *CD4^+^ T* cells. Recent research indicates that the myocardium undergoes adverse cardiac remodeling due to innate and adaptive immunity in response to cardiac pressure overload (52). Reactive oxygen species (ROS) -induced specific antigen production activates the clonal expansion and proliferation of *CD4^+^ T* cells, which ultimately leads to the infiltration of activated *CD4^+^ T* cells into the left ventricle and the production of a large number of cytokines (such as interleukin 17A), thereby promoting cardiac remodeling (53, 54). Since the kidneys of ESRD patients could not maintain blood sodium and body water homeostasis, and maintain the intermittent nature of hemodialysis treatment (three times a week), these closely related risk factors all aggravate the overload of the cardiac pressure cycle, which in turned continuously induces myocardial cell remodeling (55, 56). Moreover, the complete loss of renal function in ESRD patients, there are complex imbalances such as uremic toxin accumulation, immune system activation, reactive oxygen species production, and calcium and phosphorus metabolism disorders, which are manifested as pro-inflammatory and anti-inflammatory markers (57). Although the above phenomenon can be improved by MHD treatment, but it is not completely comparable to the filtration function of the kidney and the molecular size of the uremic toxins completely removed, will lead to residual accumulation of uremic substances (57). In addition, due to the heterogeneity of dialysate, filter membrane, and dialysis pipeline itself, it may lead to the activation of the immune system and aggravate the micro-inflammatory state (57). Based on the above research, we speculated that CDCA2, DEPDC1B and TYMS genes may promote the occurrence of HF by changing the immune inflammatory response of MHD, but this still needed further research to confirm. Based on the current research, we speculated that CDCA2, DEPDC1B and TYMS genes might be involved in the activation of the innate immune system and micro-inflammation of MHD, which in turn mediates heart and kidney terminal organ damage and eventually leads to the occurrence of HF. Future research may further clarify the internal mechanism.

Although this was a comprehensive and novel evaluation system for exploring hub genes and related signaling pathways in patients with MHD, our study also had some limitations. First of all, although we focused on the analysis of significantly up-regulated genes in this study, we recognized that down-regulated genes may be of potential importance in the development of HF in MHD patients, because many down-regulated genes may be closely related to negative regulation in pathological processes or disease remission mechanisms, which is worthy of further study. We plan to further explore whether these down-regulated genes can also show important biological significance under other experimental conditions in future studies. Secondly, since we were the first data set sequenced for patients with MHD, the sample size in our study was very limited and cannot be verified by other data sets, so further clinical investigation is needed.

## 5 Conclusion

This study utilized advanced transcriptomic techniques to explore the molecular mechanisms underlying heart failure (HF) in patients with MHD. It comprehensively and extensively analyzed the related genes and pathways. We identified four hub genes (APOBEC3B, CDCA2, TYMS, and DEPDC1B) that broaden our understanding of the molecular mechanisms and provide potential therapeutic targets for clinical diagnosis and treatment.

## Data Availability

The datasets presented in this study can be found in online repositories. The names of the repository/repositories and accession number(s) can be found in the article/Supplementary Material.

## References

[1] Liyanage T Ninomiya T Jha V Neal B Patrice HM Okpechi I Worldwide access to treatment for end-stage kidney disease: a systematic review. Lancet. (2015) 385:1975–82. 10.1016/S0140-6736(14)61601-9 25777665

[2] House AA Wanner C Sarnak MJ Piña IL McIntyre CW Komenda P Heart failure in chronic kidney disease: conclusions from a kidney disease: improving global outcomes (KDIGO) controversies conference. Kidney Int. (2019) 95:1304–17. 10.1016/j.kint.2019.02.022 31053387

[3] Damman K Valente MA Voors AA O’Connor CM van Veldhuisen DJ Hillege HL. Renal impairment, worsening renal function, and outcome in patients with heart failure: an updated meta-analysis. Eur Heart J. (2014) 35:455–69. 10.1093/eurheartj/eht386 24164864

[4] Wang F Yang C Long J Zhao X Tang W Zhang D Executive summary for the 2015 annual data report of the China kidney disease network (CK-NET). Kidney Int. (2019) 95:501–5. 10.1016/j.kint.2018.11.011 30784660

[5] Nichols GA Ustyugova A Déruaz-Luyet A O'Keeffe-Rosetti M Brodovicz KG. Health care costs by type of expenditure across eGFR stages among patients with and without diabetes, cardiovascular disease, and heart failure. J Am Soc Nephrol. (2020) 31:1594–601. 10.1681/ASN.2019121308 32487562 PMC7350988

[6] Lai AC Bienstock SW Sharma R Skorecki K Beerkens F Samtani R A personalized approach to chronic kidney disease and cardiovascular disease. J Am Coll Cardiol. (2021) 77:1470–9. 10.1016/j.jacc.2021.01.028 33736830

[7] Samanta R Chan C Chauhan VS. Arrhythmias and sudden cardiac death in End stage renal disease: epidemiology, risk factors, and management. Can J Cardiol. (2019) 35:1228–40. 10.1016/j.cjca.2019.05.005 31472819

[8] Schefold JC Filippatos G Hasenfuss G Anker SD von Haehling S. Heart failure and kidney dysfunction: epidemiology, mechanisms and management. Nat Rev Nephrol. (2016) 12:610–23. 10.1038/nrneph.2016.113 27573728

[9] Matsushita K Ballew SH Wang AY Kalyesubula R Schaeffner E Agarwal R. Epidemiology and risk of cardiovascular disease in populations with chronic kidney disease. Nat Rev Nephrol. (2022) 18:696–707. 10.1038/s41581-022-00616-6 36104509

[10] Sun CY Sung JM Wang JD Li CY Kuo YT Lee CC A comparison of the risk of congestive heart failure-related hospitalizations in patients receiving hemodialysis and peritoneal dialysis—a retrospective propensity score-matched study. PLoS One. (2019) 14:e0223336–336. 10.1371/journal.pone.0223336 31574134 PMC6773217

[11] Zhu Y Yang X Zu Y. Integrated analysis of WGCNA and machine learning identified diagnostic biomarkers in dilated cardiomyopathy with heart failure. Front Cell Dev Biol. (2022) 10:01–18. 10.3389/fcell.2022.1089915 PMC9760806 36544902

[12] Tian Y Yang J Lan M Zou T. Construction and analysis of a joint diagnosis model of random forest and artificial neural network for heart failure. Aging. (2020) 12:26221–35. 10.18632/aging.202405 33401250 PMC7803554

[13] Ma C Tu D Xu Q Wu Y Song X Guo Z Methylation in MAD1L1 is associated with the severity of suicide attempt and phenotypes of depression. Clin Epigenetics. (2023) 15:01–22. 10.1186/s13148-022-01394-5 PMC9811786 36600305

[14] Heidenreich PA Bozkurt B Aguilar D Allen LA Byun JJ Colvin MM 2022 AHA/ACC/HFSA guideline for the management of heart failure: a report of the American College of Cardiology/American Heart Association joint committee on clinical practice guidelines. Circulation. (2022) 145:e895–1032. 10.1161/cir.0000000000001063 35363499

[15] McDonagh TA Metra M Adamo M Gardner RS Baumbach A Böhm M 2021 ESC guidelines for the diagnosis and treatment of acute and chronic heart failure. Eur J Heart Fail. (2022) 24:4–131. 10.1002/ejhf.2333 35083827

[16] Zhao X Jiang L Fang X Guo Z Wang X Shi B Host-microbiota interaction-mediated resistance to inflammatory bowel disease in pigs. Microbiome. (2022) 10(115):01–22. 10.1186/s40168-022-01303-1 PMC9338544 35907917

[17] Bian Z Fan R Xie L. A novel cuproptosis-related prognostic gene signature and validation of differential expression in clear cell renal cell carcinoma. Genes. (2022) 13:851–16. 10.3390/genes13050851 35627236 PMC9141858

[18] Ming J Sana S Deng X. Identification of copper-related biomarkers and potential molecule mechanism in diabetic nephropathy. Front Endocrinol (Lausanne). (2022) 13:01–16. 10.3389/fendo.2022.978601 PMC9623046 36329882

[19] Yao H Li C Tan X. An age stratified analysis of the biomarkers in patients with colorectal cancer. Sci Rep. (2021) 11(2):064–76. 10.1038/s41598-021-01850-x PMC8599678 34789836

[20] Zhong Y Du G Liu J Li S Lin J Deng G RUNX1 and CCL3 in diabetes mellitus-related coronary artery disease: a bioinformatics analysis. Int J Gen Med. (2022) 15:955–63. 10.2147/IJGM.S350732 35115821 PMC8805863

[21] Bian R Xu X Li W. Uncovering the molecular mechanisms between heart failure and end-stage renal disease via a bioinformatics study. Front Genet. (2023) 13:001–13. 10.3389/fgene.2022.1037520 PMC9871391 36704339

[22] Gu X Lai D Liu S Chen K Zhang P Chen B Hub genes, diagnostic model, and predicted drugs related to iron metabolism in Alzheimer’s disease. Front Aging Neurosci. (2022) 14:949083–99. 10.3389/fnagi.2022.949083 35875800 PMC9300955

[23] Levin MG Tsao NL Singhal P Liu C Vy HMT Paranjpe I Genome-wide association and multi-trait analyses characterize the common genetic architecture of heart failure. Nat Commun. (2022) 13:6914. 10.1038/s41467-022-34216-6 36376295 PMC9663424

[24] Boudreau HE Broustas CG Gokhale PC Kumar D Mewani RR Rone JD Expression of BRCC3, a novel cell cycle regulated molecule, is associated with increased phospho-ERK and cell proliferation. Int J Mol Med. (2007) 19:29–39. 17143545

[25] Marchesi S Montani F Deflorian G D’Antuono R Cuomo A Bologna S DEPDC1B coordinates de-adhesion events and cell-cycle progression at mitosis. Dev Cell. (2014) 31:420–33. 10.1016/j.devcel.2014.09.009 25458010 PMC4250264

[26] Sokol S. A role for wnts in morpho-genesis and tissue polarity. Nat Cell Biol. (2000) 2:E124–5. 10.1038/35017136 10878822

[27] Yang Y Liu L Cai J Wu J Guan H Zhu X DEPDC1B enhances migration and invasion of non-small cell lung cancer cells via activating wnt/β-catenin signaling. Biochem Biophys Res Commun. (2014) 450:899–905. 10.1016/j.bbrc.2014.06.076 24971537

[28] Zhao Y Wang C Hong X Miao J Liao Y Hou FF Wnt/β-catenin signalingmediates both heart and kidney injury in type 2 cardiorenal syndrome. Kidney Int. (2019) 95:815–29. 10.1016/j.kint.2018.11.021 30770217 PMC6431558

[29] Murata M Bilim V Shirono Y Kazama A Hiruma K Tasaki M MicroRNAs as potential regulators of GSK-3β in renal cell carcinoma. Curr Issues Mol Biol. (2023) 45(9):7432–48. 10.3390/cimb45090470 37754254 PMC10529713

[30] Lopaschuk GD Karwi QG Tian R Wende AR Abel ED. microRNA-27a-3p but not -5p is a crucial mediator of human adipogenesis. Cells. (2021) 10:3205. 10.3390/cells10113205 34831427 PMC8625276

[31] Shen X Tang J Huang Y Lan X Lei C Chen H. CircRNF111 contributes to adipocyte differentiation by elevating PPAR*γ* expression via miR-27a-3p. Epigenetics. (2023) 18:2145058. 10.1080/15592294.2022.2145058 36377797 PMC9980459

[32] Da Dalt L Cabodevilla AG Goldberg IJ Norata GD. Cardiac lipid metabolism, mitochondrial function, and heart failure. Cardiovasc Res. (2023) 119:1905–14. 10.1093/cvr/cvad100 37392421 PMC10681665

[33] Lopaschuk GD Karwi QG Tian R Wende AR Abel ED. Cardiac energy metabolism in heart failure. Circ Res. (2021) 128:1487–513. 10.1161/CIRCRESAHA.121.318241 33983836 PMC8136750

[34] Wei Z Gan J Feng X Zhang M Chen Z Zhao H APOBEC3B is overexpressed in cervical cancer and promotes the proliferation of cervical cancer cells through apoptosis, cell cycle, and p53 pathway. Front Oncol. (2022) 12:864889. 10.3389/fonc.2022.864889 36249021 PMC9556651

[35] Covino DA Gauzzi MC Fantuzzi L. Understanding the regulation of APOBEC3 expression: current evidence and much to learn. J Leukoc Biol. (2018) 103:433–44. 10.1002/JLB.2MR0717-310R 29345375

[36] Tayanloo-Beik A Roudsari PP Rezaei-Tavirani M Biglar M Tabatabaei-Malazy O Arjmand B Diabetes and heart failure: multi-omics approaches. Front Physiol. (2021) 12:705424. 10.3389/fphys.2021.705424 34421642 PMC8378451

[37] Jankauskas SS Kansakar U Varzideh F Wilson S Mone P Lombardi A Heart failure in diabetes. Metab Clin Exp. (2021) 125:154910. 10.1016/j.metabol.2021.154910 34627874 PMC8941799

[38] Parvan R Hosseinpour M Moradi Y Devaux Y Cataliotti A da Silva GJJ. Diagnostic performance of microRNAs in the detection of heart failure with reduced or preserved ejection fraction: a systematic review and metaanalysis. Eur J Heart Fail. (2022) 24:2212–25. 10.1002/ejhf.2700 36161443 PMC10092442

[39] Yu L Feng Z. The role of toll-like receptor signaling in the progression of heart failure. Mediators Inflamm. (2018) 1:1. 10.1155/2018/9874109 PMC5822798 29576748

[40] Wang S Cao K Liao Y Zhang W Zheng J Li X CDCA2 protects against oxidative stress by promoting BRCA1-NRF2 signaling in hepatocellular carcinoma. Oncogene. (2021) 40:4368–83. 10.1038/s41388-021-01855-w 34103686

[41] Yu Z Zhang Y Shao S Liu Q Li Y Du X Identification of CDCA2 as a diagnostic and prognostic marker for hepatocellular carcinoma. Front Oncol. (2021) 11:755814. 10.3389/fonc.2021.755814 34660326 PMC8517522

[42] Jin WH Zhou AT Chen JJ Cen Y. CDCA2 promotes proliferation and migration of melanoma by upregulating CCAD1. Eur Rev Med Pharmacol Sci. (2020) 24:6858–63. 10.26355/eurrev_202006_21675 32633378

[43] Sun W Jin Y Wei C Xu Y Liu W Zhong J CDCA2 promotes melanoma progression by inhibiting ubiquitinmediated degradation of Aurora kinase A. Eur J Cancer. (2023) 188:49–63. 10.1016/j.ejca.2023.04.005 37196484

[44] Das S Frisk C Eriksson MJ Walentinsson A Corbascio M Hage C Transcriptomics of cardiac biopsies reveals differences in patients with or without diagnostic parameters for heart failure with preserved ejection fraction. Sci Rep. (2019) 9:3179. 10.1038/s41598-019-39445-2 30816197 PMC6395693

[45] Burke MA Chang S Wakimoto H Gorham JM Conner DA Christodoulou DC Molecular profiling of dilated cardiomyopathy that progresses to heart failure. JCI Insight. (2016) 1(6):e86898. 10.1172/jci.insight.86898 27239561 PMC4882118

[46] Kassem KM Ali M Rhaleb NE. Interleukin 4: its role in hypertension, atherosclerosis, valvular, and nonvalvular cardiovascular diseases. J Cardiovasc Pharmacol Ther. (2020) 25:7–14. 10.1177/1074248419868699 31401864 PMC6904928

[47] Doucet C Brouty-Boyé D Pottin-Clemenceau C Jasmin C Canonica GW Azzarone B. IL-4 and IL-13 specifically increase adhesion molecule and inflammatory cytokine expression in human lung fibroblasts. Int Immunol. (1998) 10:1421–33. 10.1093/intimm/10.10.1421 9796908

[48] Wilson PM Danenberg PV Johnston PG Lenz HJ Ladner RD. Standing the test of time: targeting thymidylate biosynthesis in cancer therapy. Nat Rev Clin Oncol. (2014) 11:282–98. 10.1038/nrclinonc.2014.51 24732946

[49] Krajinovic M Costea I Chiasson S. Polymorphism of the thymidylate synthase gene and outcome of acute lymphoblastic leukaemia. Lancet. (2002) 359:1033–4. 10.1016/S0140-6736(02)08065-0 11937185

[50] Hu YC Komorowski RA Graewin S Hostetter G Kallioniemi OP Pitt HA Thymidylate synthase expression predicts the response to 5-fluorouracil-based adjuvant therapy in pancreatic cancer. Clin Cancer Res. (2003) 9:416–571. 14519641

[51] Lee HS Chen M Kim JH Kim WH Ahn S Maeng K Analysis of 320 gastroenteropancreatic neuroendocrine tumors identifies TS expression as independent biomarker for survival. Int J Cancer. (2014) 135:128–37. 10.1002/ijc.28675 24347111

[52] Carrillo-Salinas FJ Ngwenyama N Anastasiou M Kaur K Alcaide P. Heart inflammation: immune cell roles and roads to the heart. Am J Pathol. (2019) 189:1482–94. 10.1016/j.ajpath.2019.04.009 31108102 PMC6717912

[53] Nevers T Salvador AM Velazquez F Ngwenyama N Carrillo-Salinas FJ Aronovitz M Th1 effector T cells selectively orchestrate cardiac fibrosis in nonischemic heart failure. J Exp Med. (2017) 214:3311–29. 10.1084/jem.20161791 28970239 PMC5679176

[54] Ngwenyama N Kirabo A Aronovitz M Velázquez F Carrillo-Salinas F Salvador AM Isolevuglandin-modified cardiac proteins drive CD4+T-cell activation in the heart and promote cardiac dysfunction. Circulation. (2021) 143:1242–55. 10.1161/CIRCULATIONAHA.120.051889 33463362 PMC7987774

[55] Loutradis CN Tsioufis C Sarafidis PA. The clinical problems of hypertension treatment in hemodialysis patients. Curr Vasc Pharmacol. (2017) 16:54–60. 10.2174/1570161115666170414120921 28413966

[56] Loutradis C Sarafidis PA Papadopoulos CE Papagianni A Zoccali C. The ebb and flow of echocardiographic cardiac function parameters in relationship to hemodialysis treatment in patients with ESRD. J Am Soc Nephrol. (2018) 29:1372–81. 10.1681/ASN.2017101102 29592914 PMC5967760

[57] Kadatane SP Satariano M Massey M Mongan K Raina R. The role of inflammation in CKD. Cells. (2023) 12:1581–601. 10.3390/cells12121581 37371050 PMC10296717

