# Supplementary figures and images for: DEPDC1B, CDCA2, APOBEC3B, and TYMS are potential hub genes and therapeutic targets for diagnosing dialysis patients with heart failure
Source: Front Cardiovasc Med. 2025 Jan 8;11:1442238. doi: 10.3389/fcvm.2024.1442238 (PMC11752391; doi:10.3389/fcvm.2024.1442238)

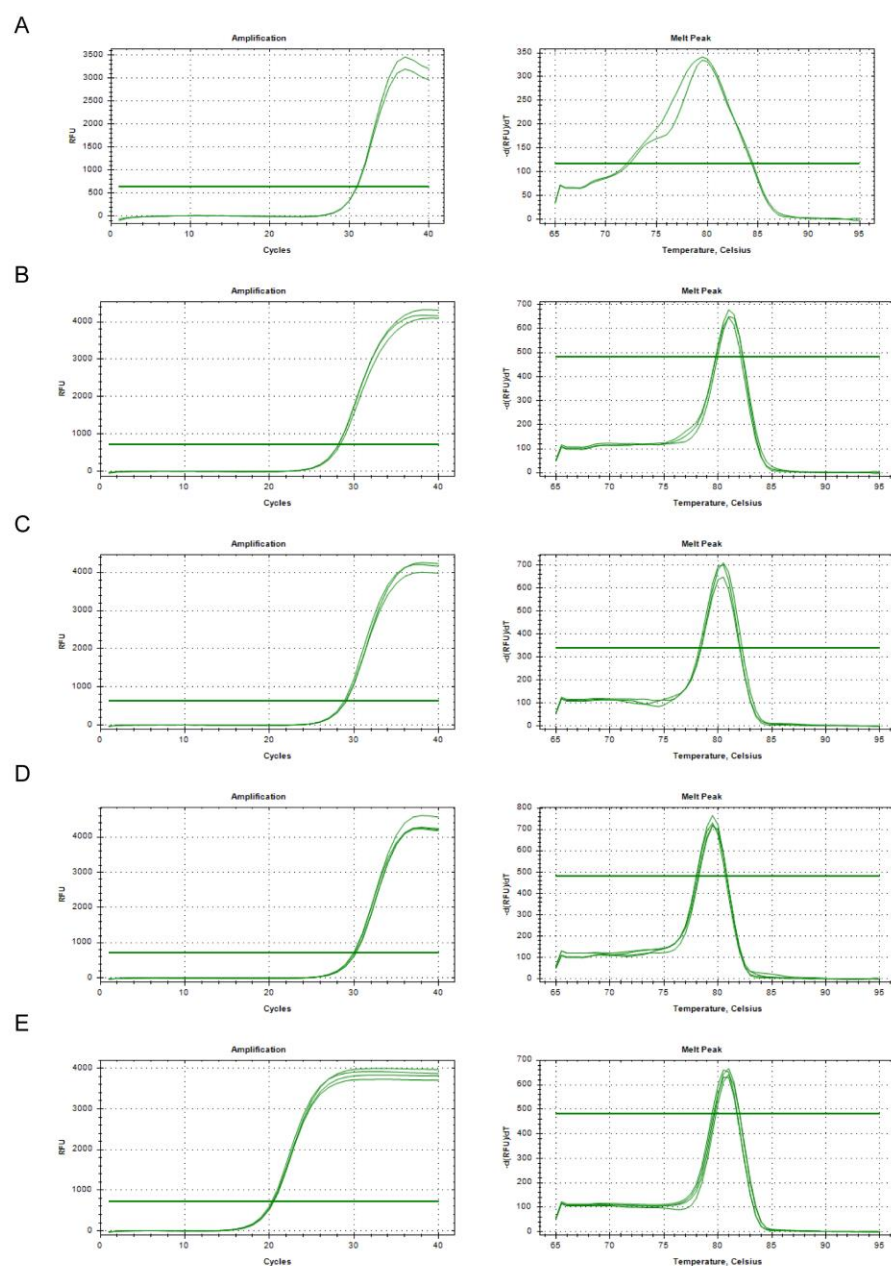

**Figure S1.** The experimental raw images of qRT-PCR

Supplement: Supplementary file 1 [file Image1.pdf]
